# Awareness and knowledge of radiation dose and associated risks among final year medical students in Norway

**DOI:** 10.1007/s13244-017-0569-y

**Published:** 2017-09-26

**Authors:** Sundaran Kada

**Affiliations:** Department of Occupational therapy, Physiotherapy and Radiography, Western Norway University of Applied Sciences, Postbox 7030, 5020 Bergen, Norway

**Keywords:** Medical education, Radiation protection, Research, Clinical practice, Students

## Abstract

**Objectives:**

The objective was to assess the knowledge of radiation dose and of the associated risks with ionising imaging examinations of medical students in their final year of training prior to graduating.

**Methods:**

An online questionnaire was sent to all final year medical students from two universities. The questionnaire consisted of radiation dose and risk related questions, with multiple choices, only one of these choices was the correct answer. A ‘correct’ answer was given one mark and no mark was given for ‘incorrect/do not know’ answers. The total mean score ranged from 0 to 11, with higher scores representing greater knowledge about radiation doses and the associated risks.

**Results:**

Ninety-nine students completed and returned the questionnaire yielding a response rate of 45%. The total mean score was 3.91 out of possible 11. Only eighteen students scored more than five points (50%). Students who reported moderate confidence in their knowledge about radiation dose and risks, scored significantly higher than students who reported no confidence (*p* = 0.003). There was a moderate positive correlation between students that reported moderate confidence and radiation knowledge scores (rho = .301, *p* = .002).

**Conclusion:**

Overall medical students’ knowledge of radiation dose and the risks associated with ionising imaging examinations was reported to be low.

**Main messages:**

• *Medical students’ knowledge about radiation and associated risk is poor*

• *Students are not aware of radiation doses for common radiological procedures*

• *The majority of students underestimated radiation doses for specific examinations*

• *Students with confidence reported greater knowledge than students with no confidence*

## Introduction

The number of medical imaging examinations that use ionising radiation in Norway is increasing. For example, between 1993 and 2002 there was an overall increase of 15% [1]. The number of CT examinations doubled (11–21%) between 2002 and 2008, and the total radiation dose from CT examinations was 79% in the year 2008 compared to 66% in 2002 [2]. X-ray radiation has dose-dependent adverse effects that lead to an increased risk of developing cancer [3]. The cumulative risk of cancer, related to the diagnostic use of x-rays, is estimated to be 1.2% by the age of 75, which translates to approximately 77 new cases of cancer per year in Norway [4, 5]. It is, therefore, the responsibility of the referring clinician to determine whether it is appropriate for a patient to undergo x-ray examinations, given the expected risks involved [6]. This judgement requires clinicians to have a clear understanding of the radiation dose and risks associated with specific imaging examinations [7].

A review of previous published studies demonstrates that health care professionals have limited knowledge about radiation dose and risks with medical imaging examinations is very low, with various health care professionals having limited knowledge about the awareness of doses and associated risks of radiation from imaging procedures [8–15]. Studies have also shown that medical students have a poor knowledge of radiation dose and its associated risks [3, 14, 16–18]. However, only two studies have been conducted in Norway concerning knowledge around radiation amongst clinicians [19, 20] and no studies have evaluated ionising radiation knowledge amongst medical students. Therefore, the aim of this study was to assess the knowledge of radiation dose and its associated risks in relation to diagnostic imaging examinations amongst undergraduate Norwegian medical students prior to graduating. It is hypothesised that final year medical students may not be aware of the radiation dose and risks associated with commonly used diagnostic imaging procedures.

## Materials and method

### Design

This was a descriptive study using a one-time survey. The current study was carried out in Norway. All medical faculties (*n* = 4) were contacted to participate in this study. Two universities declined to participate. Norwegian undergraduate medical education consists of a six-year university degree programme.

### Sample

A total of 99 undergraduate medical students, from a possible sample of 220 participated. All of the participants were in the final term of their graduating year (scheduled to graduate in 2017). Data was collected in March 2017.

### Measures

Awareness, knowledge about ionising radiation and associated risks were assessed using a questionnaire tool that has been used in previous studies with the same aim [3, 8, 16, 17]. This questionnaire, was translated from English to Norwegian using the translation-back technique. The questionnaire consists of seven questions about radiation dose and risk related issues. Assuming a single chest x-ray equals one unit of radiation, students were asked to estimate radiation doses for five of the most common imaging procedures (question 4). To capture information on whether students knew that magnetic resonance imaging (MRI) and ultrasound were non-ionising procedures, these questions were framed so that they related to specific body organs (see questions 4). All the questions were in a multiple choice format with four to six options, including a ‘do not know’ response, only one of the options was the correct answer. In the analysis, a ‘correct’ answer was assigned one mark and all other responses were assigned a zero mark. The total mean score ranged from 0 to 11, with higher scores signifying greater knowledge. Furthermore, the students were asked whether they had received any lectures about ionising radiation during their programme of study and to what degree these lectures had focussed on radiation doses and risks for common imaging examinations. The study also collected demographic information including gender, age, university, confidence in their knowledge about radiation dose; and how important is what they felt they knew about ionising radiation, as it relates to radiological investigations. Actual radiation doses were derived from current literature [21–23].

### Procedure

The heads of the medical faculty were contacted to request their consent regarding their institutions’ participation in this study. After approval, the questionnaire was modified to an online survey tool, with the link and an information letter emailed to the medical faculties; the letter requested medical faculties distribute the link and information letter to all final year medical students. The information letter to the students outlined the aim of the project, provided assurances that all information would be kept confidential, emphasised the voluntary nature of participation and informed the participants that they could access the results of the survey by contacting the author.

### Ethical considerations

The study followed the standard ethical guidelines for research conducted on students in Norway. Approvals from the Medical Research Ethical Committee and the Norwegian Social Science Data Services were not required for this study.

### Data analysis

Frequency and percentages were provided for the demographic characteristics, i.e., age and gender. Descriptive summaries (mean, standard deviation and median) were provided for the radiation knowledge score. Comparisons of radiation knowledge scores between gender (male versus female), and study places (University A versus University B) were assessed using the Mann-Whitney U test. The Kruskal-Walllis test was used to assess the difference in radiation-related knowledge scores between age categories (20–24 years, 25–29 years, 30–34 years and >34 years). Correlations between knowledge scores and the students’ confidence level in knowledge were assessed using the Spearman correlation test. A *p*-value of less than 0.05 was considered statistically significant. All statistical analyses were carried out using the statistical package for the social sciences (SPSS), version 23.0 (SPSS Inc., Chicago, IL, USA).

## Results

Seventy-five students completed and returned the questionnaire initially, yielding a response rate of 33%. The response rate was increased to 45% after sending a reminder letter. Forty-five students (45%) were male and 54 (55%) were female. Seventy-nine students (80%) were from University A and 20 (20%) were from University B. Fifty-four students (55%) reported that they were very or moderately confident in their knowledge of radiation dose; 45 students (45%) reported that they were not really confident or, ‘do not know’. Ninety-seven students (96%) reported that knowledge of radiation dose and its associated risks is very important or moderately important. The sociodemographic characteristics of the participants are presented in Table 1.

**Table 1:** Sociodemographic characteristics of participants (*n* = 99)

**Variable**	**N (%)**
Gender	
Male	45 (45)
Female	54 (55)
Age (years)	
20–24 years	10 (10)
25–29 years	74 (75)
30–34 years	11 (11)
> 34 years	4 (4)
Study place	
University A	79 (80)
University B	20 (20)
Confidence with knowledge	
Very confident	1 (1)
Moderately confident	53 (54)
Not really confident	42 (42)
Do not know	3 (3)
Perceived importance	
Very important	49 (50)
Moderately important	47 (47)
Not really important	1 (1)
Do not know	2 (2)

**Fig. 1:**
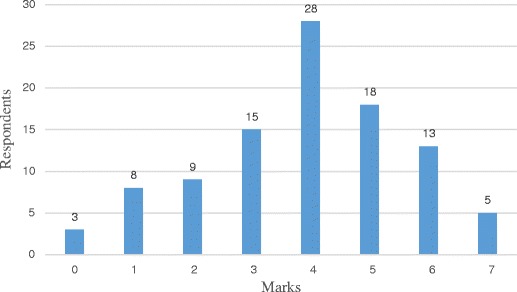
Distribution of marks scored by respondents (*n* = 99)

The average mean score was 3.91 (SD = 1.70, range 0 to7) out of possible score of 11. The average mean score for students from University A was 3.89 (SD = 1.76) and for the students from University B it was 4.00 (SD =1.49) (Table 2). Three students (3%) scored zero and only eighteen students (18%) scored more than five points (50%). The distribution of marks is shown in Fig. 1. The radiation dose received during a chest x-ray was correctly identified by 20% (*n* = 20) of the students. Only 12% (*n* = 12) of the students correctly identified the risk of fatal cancer for a CT abdomen examination and 13% (*n* = 13) correctly identified the dose limits for the patients. Nearly all students, (*n* = 93) correctly identified the group of patients who would be more sensitive to radiation dose. Seventy-three percent (*n* = 72) and 85% (*n* = 84) of the students correctly identified that both MRI and ultrasound are non-ionising imaging procedures, respectively. The number of correctly answered questions and the responses to questions regarding radiation doses for particular examinations are shown in Tables 3 and 4, respectively. Only 12 students (12%) answered correctly that there is no dose limit for patients.

**Table 2:** Mean score (SD), median and *P* values

Variable	Mean score (SD)	Median	*P* values
**Gender**			0.589^1^
Male students	4.02(1.57)	4.00	
Female students	3.81(1.81)	4.00	
**Study Place**			0.821^1^
University A	3.89(1.76)	4.00	
University B	4.00(1.49)	4.00	
**Age group (years)**			0.640^2^
Between 20 and 24	4.30(1.89)	4.50	
Between 25 and 29	3.80(1.75)	4.00	
Between 30 and 34	4.36(1.36)	4.00	
> 34	3.75(1.26)	4.00	
**Confidence of knowledge**			0.003^1^
Very or moderately confident	4.37(1.57)	4.00	
Not really confident/Do not know	3.36(1.71)	4.00	

^1^P values are conducted using the Mann Whitney U test

^2^ *P* values are conducted using the Kruskal Wallis test

**Table 3:** Frequency distributions of responses to questionnaires (*n* = 99)

Questions	Correct n (%)	Incorrect n (%)	Do not know n (%)
1. Approximately how much terrestrial radiation in millisieverts (mSV) does a person absorb in Norway in a year?	45(46)	20(20)	34(34)
2. The Norwegian population is exposed to radiation from various sources. Of these, medical radiation contributes	7(7)	65(66)	27(27)
3. Approximately how much radiation does a patient absorb during a posterio-anterior (PA) chest X-ray?	20(20)	55(56)	24(24)
4.If the exposure to PA chest radiograph is taken as one unit, how many units would a patient absorb during the following procedures?			
CT abdomen	22(22)	60(61)	17(17)
CT lumbar spine	16(16)	67(68)	16(16)
MRI brain	72(73)	10(10)	17(17)
X-ray lumbar spine	3(3)	81(82)	15(15)
Ultrasound kidneys	84(85)	15(15)	-
5.What is the risk of inducing a fatal cancer from a CT scan of the abdomen?	12(12)	60(61)	27(27)
6. What is the annual whole body dose limit for a patient?	12(12)	55(56)	32(32)
7. Which one of the following group is most sensitive to radiation?	93(94)	3(3)	3(3)

**Table 4:** Frequency distribution of responses to different level of radiation (*n* = 99)

Procedure	Number of units equivalent to a chest X-ray (a chest X-ray = 1 unit)						
	0–20	21–50	51–100	101–200	201–500	>500	Do not know
CT abdomen	1 (1%)	9(9%)	12(12%)	25(25%)	**22(22%)**	13(13%)	17(17%)
CT lumbar spine	1 (1%)	11(11%)	16(16%)	29(29%)	**16(16%)**	8 (8%)	18(18%)
MRI brain	72(73%)	5(5%)	4(4%)	-	**1(1%)**	-	17(17%)
X-ray lumbar- spine	61(62%)	16(16%)	**3(3%)**	2(2%)	2(2%)	-	15(15%)
Ultrasound -kidneys	**84(85%)**	-	-	-	-	-	15(15%)

The differences in knowledge scores between gender, universities and age group were compared. No significant differences were observed between University A and B (*p* = 0.821) or between gender (*p* = 0.589) or between age groups (*p* = 0.640).

In relation to confidence in knowledge, students who reported being moderately confident in their knowledge had significantly higher scores (mean = 4.37, median = 4.00, SD = 1.57) when compared to students who reported not being confident in their knowledge (mean score = 3.36, median = 4, SD =1.71, *p* =. 003). The Spearman correlation between students’ level of confidence in knowledge and the radiation-related knowledge score was 0.301 (*p* = .002).

The majority of the students (83%) reported that they had received lectures about ionising radiation during their study. However, only 39% (*n* = 39) of students indicated that these lectures were focussed on radiation dose and risks.

## Discussion

The aim of this study was to assess the knowledge of final year Norwegian undergraduate medical students around radiation dose and risk. To the best of my knowledge this is the first study of its kind to be conducted in Norway. The key finding of the present study is that students demonstrated low levels of knowledge (mean score 3.91 out of 11.00 [35.55% correct]). These results support the hypothesis that medical students are not aware of radiation doses and its associated risks for commonly performed diagnostic imaging procedures. The findings of this study are consistent with those of previous studies that also report poor knowledge of ionising radiation procedures amongst medical students [3, 15–18].

The mean score was 3.91 out of possible 11. It is alarming that only 20% of students correctly identified the radiation dose received during a chest X-ray examination, which is one of the most common imaging procedures performed [13]. Zhou et al. found that 31.6% of medical students were able to correctly answer this question, therefore, the students in the present study have performed less well than those in previous studies. Only 12% of students correctly responded that there was a risk of fatal cancer for CT abdomen examinations. Again this is concerning, as in Norway the number of CT examinations performed, doubled between 2002 to 2008, with these examinations contributing to 79% of the collective dose received from medical examinations in 2008 [2]. Berrington et al. (2009) estimated that in the USA, approximately 29,000 cancers developed as a direct result of CT examinations in 2007 [24], with children being more likely to develop cancer as a consequence of diagnostic imaging that uses ionising radiation. Two large scale studies have demonstrated the risks of developing brain cancer and leukaemia amongst children who underwent CT examinations [25, 26] and the lifetime cancer risks resulting from radiation exposure for children are four to five times higher than for adults [27]. Therefore, it is encouraging that 94% of the students correctly identified children as the group most susceptible to the risks associated with radiation dose.

The assessment of students’ knowledge about MRI and ultrasound yielded disappointing results with 27% and 15% of the study population not being aware that MRI and ultrasound are non-ionising procedures. This finding is similar to that of another study in Australia that found a number of students were unable to identify that MRI (25.5%) and ultrasound (11.3%) were in fact non-ionising procedures [3]. In relation to radiation dose limit for the patient, 89% (*n* = 88) of students are not aware that there are no dose limits for patients as long as the examination is justified. Although, unlike staff, there is no dose limit for patients, this does not mean that examinations can be requested carte blanche, as there is a risk associated with ionising radiation, as previously explained. Examinations need to be justified, that is, the examination should be appropriate to answering the clinical question and use a radiation dose that is as low as reasonably achievable [6]. In some cases it may be that the most appropriate examination for answering the clinical question uses non-ionising radiation, e.g., MRI or US. However, if the referring clinician is not aware of the doses of common radiological procedures, or that MRI and US utilises non-ionising radiation then there is a real possibility that inappropriate examinations are requested.

Fifty-four percent of students reported a moderate confidence in their knowledge about radiation and risks. This study, like Zhou et al. (2010) and Dellie et al. (2015) found that students who reported moderate confidence in their knowledge, scored better than those who were not confident with their knowledge [3, 14]. The present study finding is inconsistent with the earlier study [15] that identified that there was a negative relationship between students confidence and radiation related knowledge and that students who reported moderate confidence in their knowledge on radiation related knowledge scored lower scores in objective tests.

Poor knowledge and underestimation of radiation doses may lead to ionising imaging examinations being prescribed unnecessarily, resulting in an increased risk for patients. It is also apparent that this lack of knowledge will make it difficult to inform patients about the risks and benefits of a radiological examination. Ukkola et al. (2016) demonstrated that the majority of patients wanted to know about radiation dose and the risks associated with this radiation [28] and instructing patients about radiation and its effects is an integral part of the medical personnel’s responsibility. The referrer should ensure that the patient is provided with adequate information about the benefits and risks associated with the radiation dose from medical exposure prior to the examination [29]. Without this information, the patient is unable to make decisions about alternative treatments based on the advantages and disadvantages of a particular procedure.

Although the majority of students (83%) reported that they had lectures about radiation during their study, only 39% reported that these lectures focussed on radiation dose and associated risks. As the subjects are future doctors referring patients for imaging examinations, they should be taught about the approximate quantity of radiation involved and which imaging methods use radiation and which do not. Knowledgeable and well-trained students play an important role in the creation of a positive radiation safety culture. The revised Euratom basic safety standard (BSS) Directive, article 18, states that 'member states shall encourage the introduction of a course on radiation protection in the basic curriculum of medical and dental schools’ [29, p.15]. Radiation protection courses for medical students should include knowledge needed by a referring physician, i.e., basic knowledge on patient radiation protection such as biological effects of radiation, justifications of exposures, procedure optimisation, risk-benefit analysis, typical doses for each type of examination, etc. In addition, knowledge of the advantages and disadvantages of the use of ionising radiation in medicine, should be part of radiation protection education and training for medical students [30, p.14]. ICRP (2009) recommends a total of 5–10 h for radiation protection education and training for medical students [31]. However, universities, including the sampled universities, provide an average of only two hours of lectures on radiation protection and there are no learning outcomes for radiation protection lectures in the curriculum [32]. This is alarming and may reflect the attitudes of the academic staff towards radiation protection issues. It is, therefore, suggested that a radiation protection curriculum, that covers the topics and the learning outcomes that are recommended for referrers in EU Directive 2014 [30], is developed, and the number of teaching hours needs to be consistent with the ICRP (2009) recommendations [31]. Radiation safety training should be an essential part of a university’s commitment [33]. Appropriate knowledge about radiation dose and protective measures from ionising medical examinations are important components for guideline adherence [34].

## Limitations

The response rate was only 45%, and there is no explanation for this low response rate, though participation was voluntary. Response rates are often related to interest in the subject, and the poor response rate might actually be due to low interest in radiation protection. The questionnaire, despite its use in a number of studies, is not validated. The study findings, therefore, have to be interpreted with caution.

## Conclusion

The current study demonstrates that there is a low level of knowledge amongst final year medical students regardless of gender, age group and university they are attending. Students that reported a perceived importance of this topic also demonstrated significantly more knowledge than students that rated the topic as not important. The majority of students reported that the radiology component of lectures does not focus enough on radiation dose and associated risks.
